# Tick-Borne Encephalitis in Pregnant Woman and Long-Term Sequelae

**DOI:** 10.3201/eid2903.221328

**Published:** 2023-03

**Authors:** Aurélie Velay, Ralf Janssen-Langenstein, Stéphane Kremer, Elodie Laugel, Maximilian Lutz, Anne Laure Pierson, Marie-Josée Wendling, Francis Schneider, Samira Fafi-Kremer

**Affiliations:** Strasbourg University Hospital, Strasbourg, France (A. Velay, R. Janssen-Langenstein, S. Kremer, E. Laugel, A.L. Pierson, M.-J. Wendling, F. Schneider, S. Fafi-Kremer);; Université de Strasbourg, Strasbourg (A. Velay, E. Laugel, S. Fafi-Kremer);; Charité Universitätsmedizin Berlin, Berlin, Germany (M. Lutz)

**Keywords:** tick-borne encephalitis, tick-borne encephalitis virus, TBEV, pregnant woman, long-term sequelae, intensive care, ticks, meningitis/encephalitis, zoonoses, viruses, France, Germany

## Abstract

We report a case of severe tick-borne encephalitis in a pregnant woman, leading to a prolonged stay in the intensive care unit. She showed minor clinical improvement >6 months after her presumed infection. The patient was not vaccinated, although an effective vaccine is available and not contraindicated during pregnancy.

Tick-borne encephalitis (TBE), an emerging infectious disease, has shown a deeply evolving epidemiology during the past decade, especially in Europe (*1*). TBE virus (TBEV) is transmitted mainly to humans by tick bites and occasionally by consumption of contaminated dairy products (*1*). Although most infections caused by the TBEV European subtype are asymptomatic, some patients’ conditions could worsen to show severe encephalitis, associated with long-term sequelae (*1*). Data dealing with TBEV infection during pregnancy are scarce. We report a case of severe TBE and long-term sequelae in a pregnant woman.

In July 2020, a 34-year-old woman at 20 weeks of gestation was admitted to an emergency department in Strasbourg, France, because of meningismus associated with nystagmus. The patient lived in Berlin, Germany, traveled to the Black Forest (Germany), and visited Provence (southeastern France) and Alsace (northeastern France) on the way home before symptom onset.

On day 3, TBEV serologic results were positive for IgM and negative for IgG (Figure). The patient progressed to severe hyperactive delirium, requiring sedation and intubation. After a second lumbar puncture, results of reverse transcription PCR testing of cerebrospinal fluid (CSF) was positive for TBEV (Figure). A second MRI showed signs of diffuse leptomeningitis with deep cerebral nuclei involvement (Appendix Figure). At cessation of sedation (day 7), the patient remained in a coma. Iterative TBEV serologic results showed appearance of specific IgG (Figure).

**Figure:**
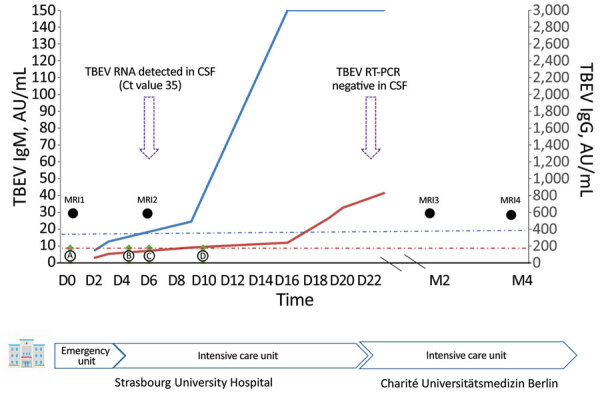
Tick-borne encephalitis in pregnant woman and long-term sequelae showing relevant clinical and laboratory findings, including TBEV antibody kinetics in serum samples. TBEV IgM (blue curve) and IgG (red curve) were detected in serum samples by using the Serion ELISA Classic TBE Virus IgG/IgM Kit (https://www.serion-immunologics.com) according to the manufacturer’s instructions. Results are expressed in arbitrary units (AU) per milliliter, with a positive threshold of 15 AU/mL for IgM (blue dot-dash line) and 150 AU/mL for IgG (red dot-dash line). Green arrows indicate clinical findings; black circles indicate timing of MRIs; purple arrows indicate TBE real-time RT-PCR performed for CSF, with the Ct value for a positive result. An in-house RT-PCR for TBEV nucleic acid was performed on CSF samples. Primer and probe sequences targeted the 3′-untranslated region of the viral genome as described by Cassinoti and Swchaiger (*2*). A positive control, a negative control, and an internal control were included to monitor overall efficiency of the RT-PCR. CSF, cerebrospinal fluid; Ct, cycle threshold; D, day after admission; M, month after admission; MRI, magnetic resonance imaging; RT-PCR, reverse transcription-PCR; TBEV, tick-borne encephalitis virus.

At admission (day 0), lumbar puncture showed pleocytosis (70 cells/mm^3^) and 55 mg/dL of protein, and all virologic molecular tests results and bacterial culture results were negative. Lyme borreliosis serologic test results were negative. Results of magnetic resonance imaging (MRI) of the brain were unremarkable. Faced with a worsening of her condition, she was transferred to the intensive care unit 2 days later and showed aseptic meningoencephalitis. Given her recent history of travel, we tested for West Nile virus, dengue virus, Zika virus, Toscana virus, and chikungunya virus; all results were negative. An autoimmune etiology was ruled out by biologic testing.

At the beginning of August 2020, the patient was transferred to Charité Universitätsmedizin in Berlin, Germany. The next 2 MRIs, performed in September and November 2020, showed progression to deep cerebral nuclei and thalamic hemorrhagic transformation and cerebral atrophy (Appendix Figure). She was discharged to a neurologic rehabilitation center after 85 days of hospitalization and had tetraparesis and polyradiculitis. A tracheostomy and a gastrostomy were performed. After intensive rehabilitation, the patient showed slow and minor clinical improvement. She was not vaccinated against TBE and did not recall either a tick bite or consumption of raw milk products. All uterine ultrasounds performed during her hospitalization showed development of the fetus on schedule. The patient gave birth to a healthy boy by cesarean delivery at term.

Six previous cases of TBEV infection occurring during pregnancy have been published (*2**,**3*). For this case, as well as for 2 previously reported cases, pregnancy proceeded normally despite severe maternal infection (*4*). However, for 2 cases reported in 1966 (*3*), premature birth and fetal or neonatal intracranial hemorrhage occurred after the mother was infected. Although vertical transmission is known to occur with other arboviruses, such as Zika virus, to date, it has not been demonstrated for TBEV in humans (*4*) and has only been described in some animal models (*5*,*6*). Transplacental transmission seems unlikely because of the barrier function of the placenta and the short time of TBEV viremia in natural infection (*1*).

Pregnancy-associated changes in the immune system probably influenced the critical state of the patient. Usually, during the phase involving central nervous system symptoms, specific TBEV antibodies appear in blood or CSF samples, but viral RNA cannot be detected in those biologic fluids. TBEV RNA is rarely detected in CSF samples, as for our patient, corresponding to severe or fatal cases occurring in immunosuppressed patients (*7*,*8*). Relative to pregnancy-related immunotolerance, this patient also showed development of a delayed humoral immune response to TBEV because the first serologic results were negative at the onset of clinical central nervous system disease (*1*,*7*).

Cellular immune response is also required for the clearance of TBEV infection (*1*,*6*,*9*,*10*). We did not explore the cell-mediated response of our patient, but it was potentially also weakened by her pregnancy condition, which could explain the prolonged viral replication in CSF.

Concordant with the severe disease progression of this patient, iterative MRI showed cerebral meningo-radiculoencephalitis evolving to deep cerebral nuclei and thalamic hemorrhagic transformation and cerebral atrophy. Abnormalities on brain MRI are reported in only 20% of TBE patients (*7*).

As for all previously reported cases, this patient was not vaccinated against TBE. However, an effective vaccine is available and not contraindicated during pregnancy.

Further research is warranted to assess the course of TBEV infection during pregnancy. In this context, our case study offers relevant information and highlights the need for vaccination against TBE in disease-endemic areas.

Appendix Additional information on tick-borne encephalitis in pregnant woman and long-term sequelae.
