# Supplementary material for: Tick-Borne Encephalitis in Pregnant Woman and Long-Term Sequelae
Source: Emerg Infect Dis. 2023 Mar;29(3):669–71. doi: 10.3201/eid2903.221328 (PMC9973690; doi:10.3201/eid2903.221328)
Supplement: Appendix — Additional information on tick-borne encephalitis in pregnant woman and long-term sequelae. [file 22-1328-Techapp-s1.pdf]

Article DOI: https://doi.org/10.3201/eid2903.221328

Tick-Borne Encephalitis in Pregnant Woman and Long-Term Sequelae

Appendix

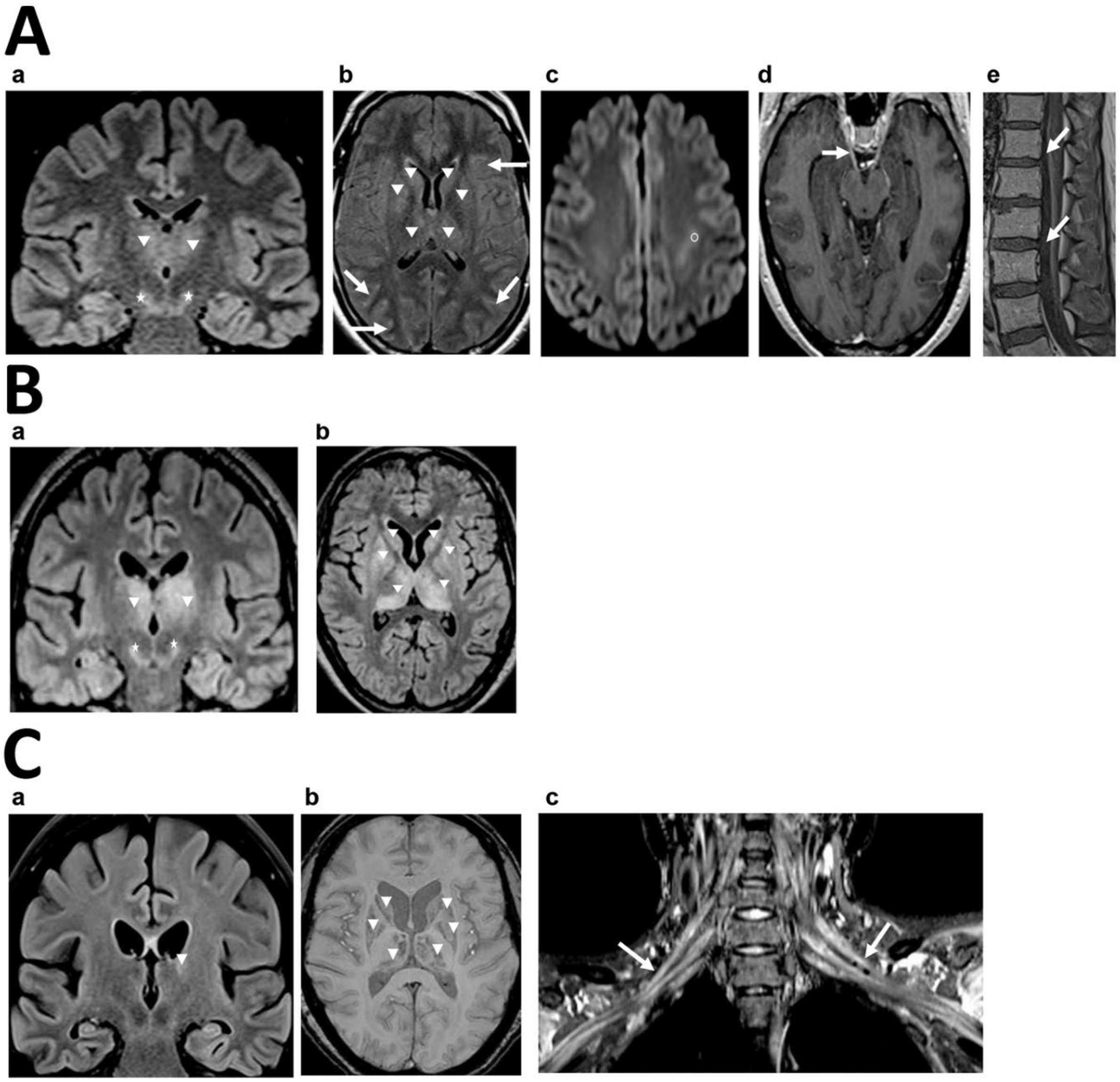

Appendix Figure. Tick-borne encephalitis in pregnant woman and long-term sequelae. Brain and spinal magnetic resonance imaging (MRI). A) Brain and spinal MRI performed at D3 (MRI2): Cerebral coronal pregadolinium fluid-attenuated inversion recovery (FLAIR) (a), axial postgadolinium FLAIR (b), axial diffusion (c), cerebral axial postgadolinium T1 (d), lumbar spine sagittal postgadolinium T1 (e). Weighted

MR images: Deep cerebral nuclei involvement with appearance of bilateral thalamic FLAIR hyperintensities (arrowheads). Crus cerebri FLAIR hyperintensities (stars). Leptomeningeal, cranial nerve and spinal nerve contrast enhancements (arrows). White matter left corona radiata diffusion hyperintensity with mild ADC decrease (white circle). B) Brain MRI performed at M2 (MRI3): Cerebral coronal (a) and axial (b) FLAIR without gadolinium weighted MR images: Persistence of deep cerebral nuclei, bilateral thalamic (arrowheads) and crus cerebri FLAIR hyperintensities (stars). Disappearance of white matter left corona radiata diffusion hyperintensity. Widening of subarachnoid spaces and enlargement of ventricles in relation to cerebral atrophy. C) Brain and spinal MRI performed at M4 (MRI4): Cerebral coronal FLAIR without gadolinium (a), axial susceptibility weighted image (b) weighted MR images: Exacerbation of cerebral atrophy. Almost total regression of deep cerebral nuclei and thalamic FLAIR hyperintensities (arrowhead), but appearance of susceptibility weighted image hypointensities corresponding to hemorrhagic transformation (arrowhead). Bilateral brachial plexus nerve enlargement and hyperintensities (arrows).
